# The Multifaceted Nature of Alexithymia – A Neuroscientific Perspective

**DOI:** 10.3389/fpsyg.2018.01614

**Published:** 2018-08-29

**Authors:** Katharina S. Goerlich

**Affiliations:** Department of Biomedical Sciences, Behavioural and Cognitive Neurosciences, University Medical Center Groningen, University of Groningen, Groningen, Netherlands

**Keywords:** alexithymia, neuroimaging, facets, dimensions, cognitive, affective

## Abstract

Neuroscientific studies have mostly employed the 20-item Toronto Alexithymia Scale (TAS-20; Bagby et al., 1994a) for the assessment of alexithymia, a self-report scale that assesses the alexithymia facets difficulty identifying feelings, difficulty describing feelings, and externally oriented thinking. These facets can be considered to capture difficulties in the cognitive processing of emotions associated with alexithymia. However, Nemiah and Sifneos’ original conceptualization of alexithymia included also an affective component, a lack of imaginative capacities, which cannot be assessed using the TAS-20. Aiming to capture the entire alexithymia construct, the Bermond–Vorst Alexithymia Questionnaire (BVAQ; Vorst and Bermond, 2001) was developed, a self-report scale which assesses two affective facets (difficulty fantasizing and difficulty emotionalizing) in addition to three cognitive facets. Based on these facets, an affective and a cognitive dimension of alexithymia can be distinguished. By now, several neuroscientific studies have investigated the neural signatures of the different facets and dimensions of alexithymia. Here, I provide an overview of the history of the alexithymia facets and dimensions and review findings provided by functional and structural magnetic resonance imaging (MRI) studies that differentiated between the alexithymia facets and/or its affective and cognitive dimensions. I then provide a synopsis of the current neuroscientific evidence for dissociable substrates of alexithymia facets and dimensions. Finally, the scientific value and clinical implications of these findings are discussed.

## Introduction

The term alexithymia was coined in 1973 by the psychotherapist Peter Emanuel Sifneos to describe patients with psychosomatic illnesses, who had several symptoms in common. These patients showed marked difficulty in identifying their feelings, in finding appropriate words to describe them, and in distinguishing feelings from bodily sensations of arousal. Moreover, they patients had little imaginative capacities, reflected in a paucity of fantasies, and a thinking style that was focused on external events, together with a striking avoidance of a focus on inner experiences. Sifneos introduced the word “alexithymia” [from the Greek *a* (no) – *lexis* (words) – *thymos* (emotion); literal meaning “no words for emotions”] to describe “this specific difficulty which appears more likely to be due to a combination of neurophysiological and psychological defects rather than to purely psychological ones.” (Sifneos, 1973).

In 1976, alexithymia was the main theme of the 11th European Conference on Psychosomatic Research held in Heidelberg, Germany (Bräutigam and von Rad, 1977). There, a consensus on the definition of the alexithymia construct was reached. Its salient features were defined as: (1) difficulty identifying feelings (DIF) and distinguishing between feelings and the bodily sensations of emotional arousal; (2) difficulty describing feelings (DDF) to other people; (3) constricted imaginal processes, as evidenced by a paucity of fantasy; and (4) a stimulus-bound, externally oriented cognitive style (Nemiah et al., 1976; Taylor et al., 1997; Taylor and Bagby, 2000). Although some individuals with alexithymia appear to contradict this definition as they can be chronically dysphoric or display sudden outbursts of weeping or rage, Taylor and Bagby (2000) note that thorough questioning usually reveals that “they know very little about their own feelings and, in most instances, are unable to link them with memories, fantasies, or specific situations.” “At the extreme, alexithymic individuals are virtually organismic automatons functioning in a one- to two-dimensional world, one that is deprived of the fullness of feelings” (Taylor et al., 1997, p. xii).

Although multiple factors are thought to play a role in the etiology of alexithymia (Nemiah, 1977), psychoanalytic theorists have mostly emphasized the contribution of early developmental deficiencies to what is referred to as *primary alexithymia* (Taylor et al., 1997). Alexithymia is considered to be primary when emerging “as a life-long dispositional factor that can lead to psychosomatic illness” (Lesser, 1981). Primary alexithymia may derive from childhood trauma (Krystal, 1979) or from negative primary caregivers interactions (Wearden et al., 2003). Moreover, the genetic polymorphism of the 5-HT transporter-linked promoter region (i.e., L/L alleles) may influence the occurrence of alexithymia (Kano et al., 2012). Hence, primary alexithymia is thought of as a more or less stable personality trait that becomes molded during childhood and early adult years, and that is therefore developmental in nature (Messina et al., 2014; see also Allen and Heaton, 2010). In contrast, secondary alexithymia refers to alexithymic characteristics resulting from developmental arrests, massive psychological trauma in childhood or later on in life, sociocultural factors, or psychodynamic factors (Taylor et al., 1997).

Clinically relevant alexithymia affects approximately ten percent of the general population (Honkalampi et al., 2001; Franz et al., 2008). Individuals with levels of alexithymia experience continuous problems processing their emotions at a cognitive level and regulating them, rendering them prone to develop psychiatric conditions characterized by affective dysregulation. Thus, alexithymia is a major risk factor for psychological distress and chronic psychopathology. Furthermore, emotion processing deficits associated with Autism Spectrum Disorders (ASD), which show high comorbidity with alexithymia, appear to be due to comorbid alexithymia rather than ASD *per se* (for a review, see Bird and Cook, 2013).

Moreover, alexithymia is linked to deficits in empathy, i.e., the ability to take the perspective of others and to understand others’ feelings and intentions. In fact, alexithymia has been found to be a transdiagnostic precursor of empathic difficulties (Valdespino et al., 2017). According to simulation theory, people simulate the feelings they observe in others to predict and understand the feelings of the people in their environment. An inability to accurately interpret and describe one’s own internal affective states will thus lead to difficulties empathizing with others’ feelings. Bird and Viding (2014) explain alexithymia-related deficits in empathy within the framework of their Self to Other Model of Empathy (SOME), whose core conceptual implication is that factors affecting one’s own experience of emotion will determine what emotional associations are learned. The authors suggest that the primary impairment in alexithymia lies within the affective representation system, which contains representations of one’s current affective state and which is likely localized to the insular cortex and the anterior cingulate cortex (ACC). Such impairment in the affective representation system would lead to an inability to form a consciously accessible representation of one’s own affective state, which is consistent with the diagnostic criterion of alexithymia as being aware of having an emotion, yet being unsure as to what emotion one experiences. In line with this, recent findings indicate that alexithymia is linked to a lack of interoceptive awareness (Mul et al., 2018), an important aspect of empathy, and that alexithymia may even be characterized by a general failure of interoception (Brewer et al., 2016; Murphy et al., 2018). However, interoceptive accuracy (a lower, physiological level of emotional awareness, which is often measured using heart beat counts) might be increased in individuals with high levels of alexithymia (Ernst et al., 2013; Scarpazza et al., 2017), in line with their tendency to overly focus on bodily signals.

Taken together, alexithymia bears major relevance for daily social and emotional functioning and for the development of psychiatric disorders and their associated societal and financial burden. Yet, even after four decades of research, the neuroscientific literature on alexithymia is undermined by disagreement regarding the operationalization and assessment of alexithymia, and by equivocal, often conflicting findings. In this article, I aim to (1) present an overview of the existing evidence for the multifaceted nature of the construct, (2) provide future directions for research into its neural substrates, and (3) discuss potential clinical implications of the presented findings.

## Alexithymia – a Multifaceted Construct

The construct alexithymia evolved an operational meaning with the development of the self-report questionnaire TAS (26-item Toronto Alexithymia Scale); which assessed four features of alexithymia: (F1) difficulty identifying and distinguishing between feelings and bodily sensations; (F2) DDF (i.e., putting feelings into words and verbalize them to others); (F3) reduced daydreaming; and (F4) externally oriented thinking (EOT) (Bagby et al., 1990). Shortly thereafter, the TAS was revised by eliminating six items assessing daydreaming, resulting in the TAS-20 (20-item Toronto Alexithymia Scale) with a three factor structure, which has become the most-widely used tool for alexithymia assessment: (F1) DIF; (F2) DDF; and (F3) EOT (Bagby et al., 1994a,b).

Despite the popularity of the TAS-20 as it provides a brief and easy-to-use tool for alexithymia assessment (which is advantageous especially in neuroimaging studies, which are usually more time-consuming and laborious than purely behavioral studies), caution is advisable.

It should be noted that DIF and DDF usually correlate highly, whereas correlations of EOT with DIF and DDF tend to be lower. Although DDF is specifically designed to capture the verbalization of feelings (i.e., the ability to find words for one’s feelings and to express one’s feelings to others), which is not explicitly part of DIF, one may argue that in order to identify a feeling, attaching a label to that feeling (in terms of inner language) is necessary. From this perspective, DIF and DDF seem relatively closely related as both explicitly refer to emotions, whereas EOT specifically assesses a style of thinking, i.e., a cognitive mode not necessarily including the experience of an emotion.

Although more objective measures of alexithymia exist, such as the observer-rated Beth Israel Hospital Questionnaire (BIQ; Sifneos, 1973), its modified version (Taylor et al., 1997), and the Toronto Structured Interview for Alexithymia (TSIA; Bagby et al., 2006), the TAS-20 provides a quick, well-validated, and standardized measure of alexithymia and has thus become the most-widely used method for its assessment. This is particularly true for neuroimaging studies as these are usually more laborious and wearisome than behavioral studies. As a consequence, the vast majority of neuroimaging studies conducted up to today relied on the TAS-20 to assess alexithymia and to shed light onto its neural basis.

Moreover, a large part of these studies used a certain TAS-20 cut-off sum score to divide participants into two groups, an alexithymic versus a non-alexithymic group or a group of high-scorers versus a group of low-scorers on alexithymia, respectively. Some studies used a cut-off score of 61, which has been suggested to indicate clinically relevant alexithymia (Taylor et al., 1988; Bagby et al., 1994b; Taylor et al., 1997). However, a number of studies used lower (and variable) cut-off scores, hampering the comparability of such studies’ findings. Importantly (regardless of the specific cut-off score applied), those studies treated alexithymia as a categorical variable and often as a unitary construct by restricting their analyses to TAS-20 sum scores. Consequently, their findings provided no insights into the neural correlates of the different facets of alexithymia. Today, however, most researchers agree that alexithymia constitutes a personality trait that is normally distributed in the population and should thus be treated as a dimensional variable rather than a categorical one. Moreover, more recent studies have come to acknowledge alexithymia as a multifaceted rather than as a unitary construct, whose facets seem to be associated with separable neural correlates. Such inconsistencies in alexithymia assessment and data analysis might have contributed to the heterogeneity in findings characterizing the alexithymia literature.

A related problem is that the “golden standard” of alexithymia assessment, the TAS-20, measures only the three abovementioned facets of alexithymia (DIF; DDF: EOT). These can be considered to capture difficulties in cognitive emotion processing in relation to alexithymia. However, Nemiah and Sifneos’ original conceptualization of the alexithymia construct included not only a cognitive but also an affective component. Nonetheless, the majority of alexithymia studies relied on the TAS-20, neglecting differences in the subjective experience of emotions. This might have further contributed to the equivocality in the literature on alexithymia.

Aiming to capture both components and thereby the complete alexithymia construct, the BVAQ was developed (Vorst and Bermond, 2001). This self-report scale assesses in addition to three cognitive alexithymia facets two affective facets: difficulty fantasizing (the degree to which a person is inclined to imagine, day-dream, etc), and difficulty emotionalizing (the degree to which a person is inclined to experience emotional feelings and to become emotionally aroused). Using the BVAQ, an affective and a cognitive dimension of alexithymia can thus be distinguished, with the cognitive dimension referring to the processing of emotions at a cognitive level (identifying, analyzing, and verbalizing feelings), and the affective dimension (fantasizing and emotionalizing) referring to the level at which an individual subjectively experiences emotions (Bermond et al., 2007). Moreover, a further differentiation between several types of alexithymia has been proposed (Bermond, 1997; Moormann et al., 2008).

## Neuroscientific Evidence for Different Facets and Dimensions of Alexithymia

Today, the idea of differentiating between different alexithymia dimensions is still considered controversial, and some empirical studies have failed to support this idea (Bagby et al., 2009; Watters et al., 2016). However, in my eyes the existing evidence suggests that such a differentiation is indeed worthwhile, for researchers and clinicians alike. Meta-analyses of functional and structural imaging studies have identified the amygdala, the insula, the ACC, and regions of the prefrontal cortex (PFC) as key correlates of alexithymia in the brain (van der Velde et al., 2013; Xu et al., 2018). However, whether these correlates are linked to specific facets and dimensions of alexithymia could not be systematically investigated due to the scarcity of evidence. Consequently, it is currently unclear whether the alexithymia facets and dimensions are linked to separable neurobiological mechanisms. Disentangling these mechanisms is critical for the development of more efficient psychological – possibly pharmacological – treatment strategies of empathy deficits and difficulties in emotion recognition and regulation associated with alexithymia. In the following, I provide an overview of the current neuroscientific evidence for separable neural substrates of the different alexithymia facets and dimensions.

## Functional Imaging

The amygdala, a key node of the emotional perception/attention system, is consistently smaller in volume and less activated during negative emotional processing in relation to higher levels of alexithymia. In two fMRI studies using masked priming paradigms, pictures of emotional (happy or sad) faces were masked with neutral faces for a very brief period of time (33 ms; milliseconds), preventing conscious recognition of the facial emotions (Kugel et al., 2008; Reker et al., 2010). Kugel and colleagues found that specifically the alexithymia facet DIF was negatively correlated with the neural response of the right amygdala to masked sad faces, even when controlling for depressivity and anxiety. Reker and coworkers reported that the TAS-20 total score and the alexithymia facets DIF and DDF significantly and negatively correlated with activation of the left amygdala in response to masked sad (but not happy) faces, controlling for trait anxiety and depression. A further study masking surprised faces with neutral ones after 33 ms found that specifically the DIF facet was negatively correlated with activity in the fusiform face area, parahippocampal gyrus and superior temporal gyrus (Duan et al., 2010).

These findings suggest that particularly the DIF facet (and to some extent also the DDF facet) of alexithymia is linked to hypoactivation in areas that are important for facial emotion processing during automatic (implicit) emotion processing. fMRI studies investigating the conscious (explicit) processing of emotions observed similar patterns of hypoactivation of the amygdala particularly for DIF in response to fear-inducing and disgusting pictures (Leweke et al., 2004) and hypoactivation of the right amygdala in response to fearful body expressions (Pouga et al., 2010). Moreover, a neurofeedback study observed that the ability of the study participants to increase their amygdala activity by recalling positive autobiographical memories was negatively correlated with DIF scores, suggesting that that the more difficulty people had identifying their feelings, the less successful they were in learning how to regulate activity within their left amygdala (Zotev et al., 2011). Taken together, these results indicate that specifically DIF is linked to a dysfunction of the amygdala (and other emotion-related areas) during the implicit and explicit processing of emotions, particularly of those with negative valence.

A PET study on hypersensitivity to bodily signals in alexithymia observed hyperactivity of the right insula and the orbitofrontal cortex (part of the PFC) during colonic distension with increasing alexithymia levels (Kano et al., 2007). Also here a difference between the alexithymia facets emerged: DIF and DDF showed similar patterns of correlation with activation in these areas, whereas EOT was related to hyperactivity in distinct (temporal) areas, which are not related to somatosensory processing. Moreover, only DIF correlated with the participants’ subjective perception during the experiment, suggesting that the more difficult it was for participants to identify their feelings, the more stressed and anxious they reported to feel, and the more intensely they experienced unpleasant sensations during colonic distension. Indeed, another study recently confirmed that primarily DIF correlates with experiences of negative affect (Suslow and Donges, 2017).

An fMRI study in patients with depersonalization disorder provided further evidence for distinct neural substrates of the alexithymia facets (Lemche et al., 2013). TAS-20 total scores correlated with neural activity in the dorsal ACC (dACC) while the patients subconsciously perceived sad facial expressions. DIF was associated with responsiveness of the anterior insula and DDF with responsiveness of the posterior cingulate cortex, both regions that are important for emotional interoception. In contrast, EOT was associated with responsiveness of the orbital gyrus, a key region of emotion regulation.

Moreover, a recent study investigating social rejection in relation to alexithymia by means of the Cyberball game observed that reduced activation in the dACC during rejection was specifically linked to the DIF facet (Chester et al., 2015). Thus, the more difficulty participants reported to have identifying their feelings, the less their dACC was activated when experiencing rejection in a social context. Moreover, DIF was the only alexithymia facet that predicted experiences of social rejection in daily life, and reduced dACC activity significantly mediated this relationship. Considering the role of emotions as feedback mechanism to guide one’s behavior, this implies that individuals scoring high on the DIF facet of alexithymia may not be able to benefit from emotional signals in terms of adapting their behavior in social contexts.

In line with the findings reported above, the results of a recent fMRI study in our lab provided evidence for distinct effects of the alexithymia facets on the processing of rewards (Goerlich et al., 2017). While participants anticipated social rewards (anticipation phase), DIF scores correlated with activity in the subgenual and perigenual ACC and the adjacent ventromedial PFC. When participants received social rewards (feedback phase), DDF scores were associated with reduced activity in the ventral tegmental area, when they received monetary rewards, DIF scores were associated with higher activity in the right insula. For EOT, no significant associations were observed, neither for (social or monetary) reward anticipation nor for feedback. These findings again highlight the specificity of the alexithymia facets on activity of the ACC, insula, and PFC during socio-emotional processing.

## Structural Imaging

A recent large-scale (*n* > 1,600) VBM study corroborated the notion of neural separability of the alexithymia facets (Grabe et al., 2014). Controlling for levels of anxiety and depression, reduced dACC volume was identified as the major structural correlate of alexithymia as assessed by TAS-20 total scores. Regarding the TAS-20 factors, the most prominent contributions to volume reductions were found in relation to DIF scores, which were linked to smaller volumes of the dACC, left middle and inferior temporal gyrus, and cerebellum. DDF scores were associated with less gray matter in the fusiform gyrus, inferior temporal gyrus, and left cerebellum. In contrast, EOT scores showed no association with gray matter volumes. These findings demonstrate that the alexithymia facets are associated not only with differences in the function of emotion-related brain regions, but also to differences in their structure.

In a VBM study of our lab, we investigated for the first time whether the cognitive and affective alexithymia dimensions could be differentiated at the structural brain level (Goerlich-Dobre et al., 2014). We found that TAS-20 total scores (indicative of the cognitive alexithymia dimension) were linked to more gray matter volume in the right posterior insula. In contrast, the affective dimension, specifically the emotionalizing factor of the BVAQ, was related to more volume in the right middle cingulate cortex. In a VBM study purely based on the BVAQ, the cognitive dimension was associated with reduced dACC volume, and the affective dimension with reduced volume of the orbitofrontal cortex (van der Velde et al., 2014). While the results of the two studies are different (probably due to differences in alexithymia assessment and performed analyses), they do suggest that the cognitive and affective alexithymia dimensions are linked to dissociable structural profiles. However, given the relatively small sample sizes of both studies (40 and 55 participants, respectively), their results should be interpreted with caution. In a further VBM study including 125 participants, using the BVAQ for alexithymia assessment, we found that the cognitive dimension was associated with volume reductions in the left amygdala, left insula, thalamus, caudate, hippocampus, and parahippocampal gyrus, whereas the affective dimension was linked to volume reduction in the middle cingulate cortex only (Goerlich-Dobre et al., 2015).

## Conclusion

Taken together, the currently available evidence suggests that the different facets and dimensions of alexithymia are indeed related to differences in the function and structure of the key correlates of alexithymia. Especially the evidence regarding the three TAS-20 factors seems fairly robust. DIF is associated with impairments in the explicit and implicit processing of facial and bodily expressions of emotions, somatosensory processing, and reward processing. One large VBM confirmed a special role of DIF particularly on volumes of the dACC, a key region for emotional self-awareness. DDF seems to affect somatosensory and facial emotion processing as well, but to a lesser extent. In contrast, EOT appears to show little effect on the function and structure of brain regions involved in emotion processing. However, it should be kept in mind that EOT refers more to a cognitive level of emotion processing, whereas DIF and DDF are (1) closely related, and (2) refer to the subjective experience of emotions, i.e., the affective level of emotion processing. This difference might explain why MRI studies focusing on emotion-related areas in the brain usually found correlations with DIF (and, to a lesser extent, with DDF), but rarely with EOT.

The evidence regarding the structural correlates of the cognitive and affective alexithymia dimensions is currently less clear. While VBM studies have provided evidence for different structural correlates of the alexithymia dimensions, their results are heterogenous. This may be due to differences in alexithymia assessment and sample sizes between studies. It should also be noted that imaging research on alexithymia has heavily relied on self-report scales, which inherently lack objectivity. Further, it could be a consequence of the persisting uncertainty regarding the validity of the two alexithymia dimensions as based on the BVAQ, especially regarding the inclusion of an emotionalizing factor in the affective dimension (Bagby et al., 2009; Watters et al., 2016).

It is thus recommendable that future studies into the multifaceted nature of alexithymia use a multimethod assessment approach as well as large sample sizes to ensure sufficient statistical power. Moreover, it will be important to control for constructs that are closely related to alexithymia, such as negative affectivity, depression, and anxiety, to reach a better understanding of the neural mechanisms that underlie alexithymia and its different facets and dimensions. Such research could have far-reaching clinical implications. In addition to reflecting a fundamental deficit in recognizing and regulating one’s own emotions, alexithymia might be associated with a lack of interoceptive awareness, interrupting the process of simulating the emotions of others in order to empathize with them. Empathy deficits are characteristic of a multitude of psychiatric conditions, including borderline personality disorder, psychopathy, narcissistic personality disorder, and ASD. Given the transdiagnostic significance of these problems and their apparent links to specific facets and dimensions of alexithymia, being able to identify their neural markers could substantially improve the neuropsychiatric assessment of people who are at risk of such disorders, and contribute to the development of individually tailored and thus more effective treatment strategies.

## Author Contributions

The author confirms being the sole contributor of this work and approved it for publication.

## Conflict of Interest Statement

The author declares that the research was conducted in the absence of any commercial or financial relationships that could be construed as a potential conflict of interest.
